# Effectiviness and safety of antidepresants in bipolar depression

**DOI:** 10.1192/j.eurpsy.2022.1851

**Published:** 2022-09-01

**Authors:** T. Jupe, B. Zenelaj

**Affiliations:** 1 Psychiatric Hospital of Attica, 5th Acute Psychiatric Department, Chaidari, Greece; 2 National Center for Children Treatment and Rehabilitationn, Child Psychiatry, Tirana, Albania

**Keywords:** Antidepressants, bipolar depression

## Abstract

**Introduction:**

Depressive episodes are associated with higher morbidity, mortality (mostly suicidality. Despite the high prevalence and the devastating impact of this condition, there is a long‐standing debate about its treatment, particularly about the use of antidepressants. International guidelines and expert consensus recommend to avoid AD for bipolar depression, or to use AD with caution and as second line treatment only if the depressive episode shows poor response to mood stabilizers (MS) and to some second generation antipsychotics (SGA) (cariprazine, lurasidone, quetiapine and olanzapine combined with fluoxetine) in monotherapy and in combination. Contrary to the advice of guidelines and experts, 50%–80% of acute bipolar depressive episodes are treated with AD in everyday clinical practice.

**Objectives:**

To evaluate the effectiveness and the safety of AD acute treatment in patients with bipolar depression

**Methods:**

Literature review (PubMed)

**Results:**

Short‐term safety, switching and suicidality did not differ significantly, and no suicide attempt was observed. Concerning long‐term safety, patients with bipolar depression had a significant reduction of depressive and total recurrences during the first year.

**Conclusions:**

Acute AD treatment of bipolar depression is effective in the short term and safe in the short‐ and long‐term

**Disclosure:**

No significant relationships.

